# Person- and job-specific factors of intuitive decision-making in clinical practice: results of a sample survey among Hungarian physicians and nurses

**DOI:** 10.1080/21642850.2020.1741372

**Published:** 2020-03-23

**Authors:** Gabor Ruzsa, Csenge Szeverenyi, Katalin Varga

**Affiliations:** a Doctoral School of Psychology, Institute of Psychology, Eötvös Loránd University, Budapest, Hungary; b Department of Statistics, School of Economics, Corvinus University of Budapest, Budapest, Hungary; c Department of Orthopedic Surgery, Medical School and Health Science Center, University of Debrecen, Debrecen, Hungary; d Department of Affective Psychology, Institute of Psychology, Eötvös Loránd University, Budapest, Hungary

**Keywords:** Intuition, clinical decision-making, medical specialties, clinical expertise, medical education, complexity, emergency

## Abstract

**Objective:** To assess the prevalence of intuitive decision-making (IDM) among health care practitioners (HCPs) and explore its person- and job-specific factors.

**Design and Outcome Measures:** We used on-line survey data from a cross-sectional sample of Hungarian physicians and nurses (*N* = 460) to assess their reliance on IDM. In a second survey we asked physicians (*N* = 104) to rate medical specialties on dimensions of ‘emergency’ (necessity of making instantaneous decisions in unforeseeable situations) and ‘complexity’ (necessity of considering multiple perceptual and diagnostic aspects of patients’ health condition along with diverse treatment options).

**Results:** Altogether 40% of participants reported ever relying on IDM. Using logistic regression analysis, we found the estimated probability of IDM was 0.24 greater for physicians than for nurses, 0.10 greater for females than for males, and 0.11 greater for advanced level HCPs than for novices. Reaching expert level further increased (by 0.31) the probability of IDM for physicians, but not for nurses. Concerning physicians, practicing in a medical specialty of ‘high likelihood of emergency’ or ‘high complexity’ increased the probability of IDM by 0.25 and 0.23; the same effects for nurses were 0.20 and 0.07. We found some (inconclusive) evidence for education positively influencing HCPs’ propensity for IDM. Additionally, we performed content analysis of participants’ free-text answers to explore the psychological background of IDM instances. HCPs educated in the subject of IDM were found more disposed to perform or request further medical investigation, less prone to deviate from medical protocols, apter to reflect on their mental processes, and more inclined to rely on a large scope of information for their decisions.

**Conclusions:** The associations between job- and person-specific factors and HCPs’ propensity for IDM may have implications for their training and allocation in the health care system. Education has great potential for enhancing the quality of IDM in clinical practice.

## Introduction

The use of intuition in clinical decision-making (CDM) has been the object of a long standing debate. Theoreticians’ attitudes to intuitive reasoning have ranged from condemnation as irrational and unreliable (Lamond & Thompson, 2000; Pellegrino, 1979) to advocacy of its positive contributions to diagnostic and treatment outcomes (Brush, Sherbino, & Norman, 2017; Green, 2012; McCutcheon & Pincombe, 2001). Despite ample evidence for health care practitioners’ (HCPs) reliance on intuitive and heuristic decision-making (Melin-Johansson, Palmqvist, & Ronnberg, 2017; Miller & Hill, 2018; Pretz & Folse, 2011; Woolley & Kostopoulou, 2013), these practices were traditionally mistrusted (Benner & Tanner, 1987; Rew & Barrow, 1987), as they were considered a deviation from sound analytical reasoning.

Research conducted in the past 30 years has provided insight to the nature of intuitive decision-making (IDM) and its role in health care. Yet, many issues need further exploration, including the prevalence of intuitive practices across different medical fields and occupations, and the role of intra-personal factors in the beneficial use of IDM.

### Intuition as a way of human information processing and decision-making

From a cognitive psychological approach, intuition is a non-analytical way of human information processing, which operates through complex pattern recognition and quick, non-conscious associations made on the basis of experiential knowledge (Epstein, 2011). The outcome of these processes is often conveyed by a subtle affective signal (Epstein, 2011; Hodgkinson, Langan-Fox, & Sadler-Smith, 2008): either a sense of reassurance (‘everything fits together’) or a sense of alarm (‘there is something wrong’) (Stolper et al., 2011). As these processes operate in the non-conscious background of cognition, intuitive insight seems to appear out of nowhere, and usually the person finds it difficult to formulate verbally how he/she has arrived at it (Hodgkinson et al., 2008; Hogarth, 2005).

This sense of mysteriousness is probably one of the reasons why conservative fields such as medicine were for a long time largely mistrusting of intuitive judgment. However, by now IDM has been acknowledged as a viable and efficient way of problem solving in a variety of domains and circumstances. This is particularly true for volatile and unpredictable situations in which there is a general lack of time and information, precluding the use of regular, normative decision-making (DM) processes (Agor, 1986; Okoli & Watt, 2018).

The role of intuition in human cognition is often considered in terms of a dichotomy between analytical and non-analytical systems, as formulated by various versions of dual-processing theory (Epstein, 1994, 2011; Evans, 2008). Analytical (‘system 2’) processing involves the conscious application of abstract logical rules to information, and as such it is slow and cognitively demanding. Non-analytical (‘system 1’) processing, on the other hand, relies on the unconscious recognition of patterns and matching them with configurations previously encountered by the individual. In contrast with system 2, non-analytical processes are fast, automatic, and effortless.

Besides intuition, heuristic judgment is another type (or rather strategy) of non-analytical information processing, which narrows the focus of perception to a handful of relevant cues with the goal of obtaining a satisfying outcome in a quick and cognitively efficient way (Gigerenzer & Gaissmaier, 2011). Although intuitive and heuristic judgment is ubiquitous in CDM, their role and appropriateness has been a debated issue.

### The role and appropriateness of intuition in clinical decision-making

Concerning the appropriateness of intuition in CDM, opposing views have been formulated by proponents of different paradigms of clinical reasoning (CR). Traditionally the most influential of these, the hypothetico-deductive and the evidence-based models don’t leave much room for intuition, as they are committed to the orthodox methods of scientific reasoning: statistical/inductive and analytical/deductive inference (Patel, Arocha, & Zhang, 2005).

The evidence-based model builds on decision theory, statistical methods, and the principles of scientific evaluation for defining best practice guidelines. For diagnosing it relies on large-scale clinical epidemiological knowledge, which (in theory) can be compared and combined with the patient’s symptoms and examination results in a way to produce a set of ‘objective’ probabilities for all conceivable diagnoses. For treatment decisions it relies on the systematic evaluation of pieces of scientific evidence about the efficacy and the risks of various treatment methods. This approach, which in its pure form is more of a normative ideal than a real-life possibility, leaves no room for intuition or any other form of non-analytical reasoning; all these are considered a deviation from the best practice.

A more realistic approach to CR is presented by the hypothetico-deductive model (Elstein, Shulman, & Sprafka, 1978). It proposes a general scheme whereby first a number of diagnostic hypotheses are generated on the basis of initial cues about the patient’s condition, and then the validity of these hypotheses is tested by verifying whether the specific predictions following from each of them are consistent with information acquired through additional measurements. This scheme allows an implicit role for intuitive judgment, as arguably the generation of diagnostic hypotheses occurs through an automatic, non-analytical pattern-matching process between the currently observed cues and the collection of medical cases previously encountered by the clinician (Elstein, 2009; Norman & Brooks, 1997; Stolper et al., 2011).

Dual-processing models of CR (Croskerry, 2009; Marcum, 2012; Pelaccia, Tardif, Triby, & Charlin, 2011) designate a larger role for IDM, which can not only serve for generating initial hypotheses but also for governing the whole diagnostic process. In routine cases, in which salient features of the patient’s symptom presentation stand out clearly and a coherent pattern is recognized, expert clinicians tend to make cognitive shortcuts to reach a conclusive diagnosis. Nonetheless, the slow and effortful analytical/deductive reasoning procedure may be necessary in more complicated cases.

Clinicians’ pattern recognition – identifying recurrent patterns in patients’ illness presentations on the basis of previous experiences and other contextual information – is universally acknowledged for its important role in clinical practice, and some theoreticians (e.g. Marcum, 2012) even consider it a standalone model of CR. Within the hypothetico-deductive and the dual-processing models, the primary role of pattern recognition lies in the automatic, experience-based generation of diagnostic hypotheses through activation of ‘system 1’ processing. However, the scope of intuition in CDM extends well beyond automatic pattern recognition, as it often involves other cognitive tendencies such as preconscious affective dispositions (Stolper et al., 2011) and insights gained through emotional attunement to the patient (Hutchinson, Hurley, Kozlowski, & Whitehair, 2018), all of which is now recognized as having potential benefits.

CDM skills based on individual experience, including the use of intuition, are now widely considered complementary to clinical practice guidelines, decision algorithms, and other advanced techniques (such as computerized clinical decision support systems) best embodying the scientific approach of evidence-based medicine. Certainly, such algorithmic methods provide great benefits; yet, as several authors (e.g. Tonelli, 1998) have pointed out, there are important limitations to their applicability. For one thing, clinical guidelines are based on epidemiological data, i.e. incidence and outcome patterns for a set of prototypical cases of medical conditions observed in the reference population. But they can only be imperfectly applied to the individual patient, who may in several respects be atypical, and whose condition may be entangled by idiosyncrasies and comorbidities.

For another thing, the evidence-based model of CDM assumes unlimited cognitive/computational capacity as well as instantaneous access to a complete and up-to-date clinical epidemiological knowledge base. In reality, canonical epidemiological knowledge is lagging many years behind public health trends and recent developments in medical research. And even if all the most up-to-date information required to find the ‘optimal’ decision for a particular case might ‘somewhere’ (within the repositories of human scientific knowledge) exist, accessing it would in many cases require prohibitively long time and/or unaffordable computational resources.

For these reasons, practitioners in most fields of medicine are likely to encounter situations in which they cannot follow clinical guidelines to the letter, and at best they can hope to work out a synthesis between the ‘available scientific evidence’ and the specific needs of the patient. So while respecting the evidence base can prevent errors due to unfounded beliefs and personal biases, clinicians’ individual, experience-based knowledge and CDM skills are essential for adapting their decisions to each medical instance.

A more radical position asserts that relying solely on evidence-based evaluation and analytical reasoning is impracticable as a method of CDM (Bodemer, Hanoch, & Katsikopoulos, 2015; Feufel & Flach, 2019; Stolper et al., 2011). This view is supported by evidence showing that simplified, heuristic ways of DM in real-life situations often outperform more complex, analytical methods (Gigerenzer & Gaissmaier, 2011; Marewski & Gigerenzer, 2012). Adopting Simon’s (1956) ecological rationality paradigm, Bodemer et al. (2015) and Marewski and Gigerenzer (2012) argue that heuristic methods are adaptive under the usual conditions of CDM, which are: inherent unpredictability, incompleteness of information, and limited time and capacity for information processing. Proponents of this approach advocate that the applicability of non-analytical CDM techniques should be investigated, those found viable should be described and further refined, and their usage should be taught to medical students (Bodemer et al., 2015; Feufel & Flach, 2019).

### The prevalence and the factors of intuitive decision-making in medicine

As it seems, there is some degree of consensus about the appropriateness of multiple ways of DM in medicine. Most theoreticians agree that both analytical and non-analytical methods have their benefits and their limitations, and using a combination of the two yields better results than relying solely on one way of CDM (Elstein, 2009; Marcum, 2012; Norman, Young, & Brooks, 2007). As intuitive and heuristic techniques are essential for narrowing down the initially unstructured decision problem (Brush et al., 2017; Elstein, 2009), their use in medical diagnosis and treatment is now accepted as a matter of fact.

Yet, surprisingly little research has been conducted about the prevalence and the factors of IDM in medicine. It is reasonable to assume that HCPs with different educational and professional backgrounds, working in a variety of medical fields and occupational positions also differ as to how much they rely on intuition in their practice. Then, which are the relevant factors of IDM? In what way and to what extent do they promote HCPs’ use of intuition? Might it be possible to improve the quality of diagnostic and treatment decisions by matching the job-specific factors calling for IDM with the person-specific factors facilitating the appropriate use of intuitive techniques? These are some of the questions which we have sought to investigate.

## The current research

The aim of our research was to explore the prevalence of IDM in clinical practice, investigate its psychological background, and identify the major factors influencing HCPs’ propensity to rely on intuition in their work. Thus, our purpose was threefold: (1) we wanted to verify whether the well-established facts about certain personal and situational factors being conducive to IDM are also valid in the context of medicine; (2) we wanted to categorize medical specialties as for the extent to which they call for IDM; (3) we sought to explore HCPs’ behavioral and cognitive tendencies involved in instances of IDM.

### A classification of medical specialties

It is reasonable to assume that medical fields are different as to how much they can accommodate IDM practices. One approach to exploring this issue would be to survey medical specialties on either the relative frequency of decisions made in an intuitive way or the proportion of HCPs relying on IDM in their work. We have adopted another approach: identify a number of qualitative dimensions predictive about the extent to which IDM practices are used in distinctive medical fields. We propose a two-way classification in terms of ‘*likelihood of emergency’* and ‘*complexity’*, two dimensions supposedly influencing the extent to which medical specialties are conducive to IDM. To our knowledge, this classification is novel in the literature.

As for the first dimension, we consider to be ‘of high likelihood of emergency’ those specialties in which unforeseeable situations demanding immediate medical intervention arise frequently. Because time pressure and rapidly changing conditions are prominent aspects of situations in which IDM is beneficial (Agor, 1986; Okoli & Watt, 2018), it is justified for a hypothesis that specialties with high likelihood of emergency should call for IDM more often and more extensively than specialties with relatively low likelihood of emergency.

As for the second dimension, we conceptualize as ‘highly complex’ those medical specialties which require a highly integrative approach to dealing with patients. That is, HCPs employed in a ‘highly complex’ specialty need to be attentive to a multitude of perceptual and diagnostic aspects of their patients, and also they need to consider a variety of treatment options along with their possible effects. This definition is in line with Zavala, Day, Plummer, and Bamford-Wade’s (2018) discussion of ‘case complexity’, an aspect of medical fields which arguably has a strong influence on CDM: ‘This variability (…) requires the integration of multiple sources of information: signs, symptoms, test results, treatments and the range of possible outcomes’ (p. 399).

We hypothesize that IDM occurs more often and on a larger scale in ‘highly complex’ specialties than in specialties of relatively low complexity, which are focused on restricted areas of patients’ biological functioning. We base this on research indicating that highly complex problems and situations often call for non-analytical ways of DM (Gigerenzer & Gaissmaier, 2011; Hogarth, 2005). Indeed, several authors have pointed out that the benefits of intuition as a mode of human cognition lie in its capacity to transcend the boundaries of analytical thinking as for the maximal complexity of problems that can be formulated and solved in an efficient way (Dreyfus & Dreyfus, 1986; Hogarth, 2005; Mero, 2002).

Having proposed these two dimensions, one of our objectives was to establish an empirical classification of medical specialties in terms of ‘complexity’ and ‘likelihood of emergency’ and confirm our hypotheses about how they relate to the prevalence of IDM.

### Research hypotheses

Building on prior research, we formulated the following hypotheses about some of the possible factors of IDM in medicine. (1) HCPs’ propensity for IDM increases with professional experience. (2) Higher responsibility health care occupations (physician) are more conducive to IDM than lower responsibility occupations (nurse). (3) The more extensive education a HCP has received in the topic of IDM, the more likely he/she will use it in his/her practice. (4) HCPs occupied in specialties of high likelihood of emergency have a greater propensity for IDM than those occupied in specialties of lower likelihood of emergency. (5) HCPs occupied in specialties of high complexity have a greater propensity for IDM than those occupied in specialties of lower complexity.

## Methods

We administered two on-line surveys to HCPs practicing in Hungary. The objective of the first survey was to assess the prevalence of IDM across fields and occupations in medicine and to examine certain behavioral and cognitive aspects of IDM in clinical practice. The second survey was aimed at rating medical specialties on the dimensions of ‘complexity’ and ‘likelihood of emergency’. As for the data analysis, we used, besides basic descriptive statistical tools, logistic regression modeling to quantify the influence of personal and occupational factors on HCPs’ propensity for IDM.

### Survey on intuitive decision-making in medicine

In 2014, we conducted our first survey, whereby a sample of Hungarian HCPs, employed in a variety of medical fields and occupations, were asked the following questions:

- Do you ever rely on intuition in your practice, i.e. do you ever make a decision which you feel right about but for which you cannot find a purely rational justification? (Yes / No)
- If yes, please describe briefly one such situation, along with the way in which you came to a decision.
- Was the topic of intuitive decision-making in medicine ever discussed in your formal studies or at vocational trainings? (No, never / Scarcely / Yes, extensively)

Besides these three questions and basic demographic characteristics, participants were asked to provide information about their occupation in health care, their medical specialty, and the number of years of professional experience they had had.

Subjects were recruited through chain-referral sampling, whereby HCPs participating in the research were asked to refer the on-line survey to their colleagues and acquaintances. Acknowledgedly this sampling method may give rise to certain biases due to its uncontrolled nature and its lack of representativeness; nonetheless, it proved to be an efficient way of obtaining a large and rather diverse sample.^1^

We received a total number of 728 responses. After the removal of mistakenly submitted duplicates and the exclusion of subjects with missing data, 667 valid entries remained in the data set. We further restricted the sample to physicians and nurses (460 altogether), discarding the data of 207 HCPs belonging to other occupations (paramedics, physiotherapists, medical assistants, midwives, health visitors, etc).

Additionally to evaluating physicians’ and nurses’ responses to the categorical survey questions, we also processed the free-text accounts of situations involving IDM. Out of the 229 case descriptions submitted by 667 HCPs in the unrestricted sample, we identified 140 as containing important characteristics of intuitive information-processing and/or intuitive decision-making. These were further were categorized as relating to diagnosis (76 accounts), treatment (51 accounts), or both (13 accounts), and then used as material for content analysis.

### Survey on the characteristics of medical specialties

In 2016, we administered our second on-line survey to a sample of Hungarian physicians in a variety of medical fields, asking them to rate 27 medical specialties on the dimensions of ‘complexity’ and ‘likelihood of emergency’. Participants were asked to give each specialty a rating from 1 to 5 in response to the following questions:

- To what extent is a complex/integrative approach to dealing with patients necessary, requiring the consideration of multiple perceptual and diagnostic aspects along with a variety of treatment options?
- How often do unexpected situations demanding immediate medical intervention arise?

Survey participants were physicians sampled from a public on-line registry representing the full variety of medical fields. We received responses from 150 out of 664 physicians contacted. As a filter criterion we imposed a minimal response rate of 75%, requiring a valid answer to have been given to at least 40 out of 54 questions. This resulted in a reduced sample of 104 physicians, whose ratings we used for the data analysis.

## Results of the survey of medical specialties

As the ultimate reason for conducting our second survey was to classify HCPs’ medical specialties as of high / low complexity and emergency, we restricted the analysis to 20 (out of 27) specialties with non-zero frequencies in the first dataset. For each medical specialty we calculated the sample mean and the sample standard deviation of ratings on both dimensions (see Table 1).

**Table 1.** Physicians’ rating (1–5) of medical specialties as for how likely emergency situations are to occur and how complex an approach to dealing with patients is required. Last two columns: distribution of physicians practicing in Hungary across specialties, compared with the sample distribution.

Medical specialty		‘Likelihood of emergency’ rating		‘Complexity’ rating		Distribution of physicians	
		mean	st. dev.	mean	st. dev.	sample	population
**Group 1**	Surgery	4.13	0.75	3.60	0.88	9.1%	10.5%
	Accident surgery	4.59	0.72	3.48	1.03	2.2%	2.6%
	Emergency medicine	4.74	0.68	4.19	1.06	7.4%	1.7%
	Intensive care medicine	4.58	0.84	4.34	1.03	3.0%	5.2%
**Group 2**	Anesthesiology	3.75	0.91	4.14	0.98	3.5%	
	Internal / general medicine	3.60	0.86	4.54	0.68	16.5%	18.7%
	Obstetrics & gynecology	3.60	0.91	3.96	0.86	4.3%	5.8%
	Neonatology	3.20	0.91	3.94	0.99	0.0%	0.8%
	Neurology	3.31	0.95	3.93	0.95	2.2%	2.8%
	Psychiatry	3.05	0.91	4.08	1.14	3.5%	3.7%
	General practice	2.78	0.72	4.25	0.80	15.2%	16.9%
	Pediatrics	2.81	0.84	4.33	0.80	11.3%	9.9%
	Geriatrics	2.59	0.75	3.96	0.94	0.0%	0.3%
**Group 3**	Oncology	2.86	0.89	3.65	0.79	3.0%	1.3%
	Physiotherapy	1.66	0.73	3.33	1.04	0.4%	0.1%
	Otolaryngology	3.00	0.82	3.03	0.85	0.4%	2.6%
	Ophthalmology	2.34	0.82	2.73	0.90	3.5%	3.2%
	Dentistry	2.24	0.85	2.48	0.95	6.5%	8.5%
	Dermatology	1.97	0.71	2.91	1.02	2.6%	2.2%
	Diagnostic imaging & laboratory diagnostics	2.07	0.96	2.22	1.05	5.2%	3.3%

On the dimension of ‘likelihood of emergency’ mean ratings ranged from 1.66 to 4.74, with an average of 3.14 across the 20 specialties. We computed an intraclass correlation coefficient *H* = 0.73, indicating a high level of inter-rater agreement on this aspect of medical fields. On the dimension of ‘complexity’ mean ratings ranged from 2.22 to 4.54, with an average of 3.57 across the 20 specialties. The intraclass correlation value was *H* = 0.57, indicating a medium / acceptable level of inter-rater agreement.

**Figure 1.**
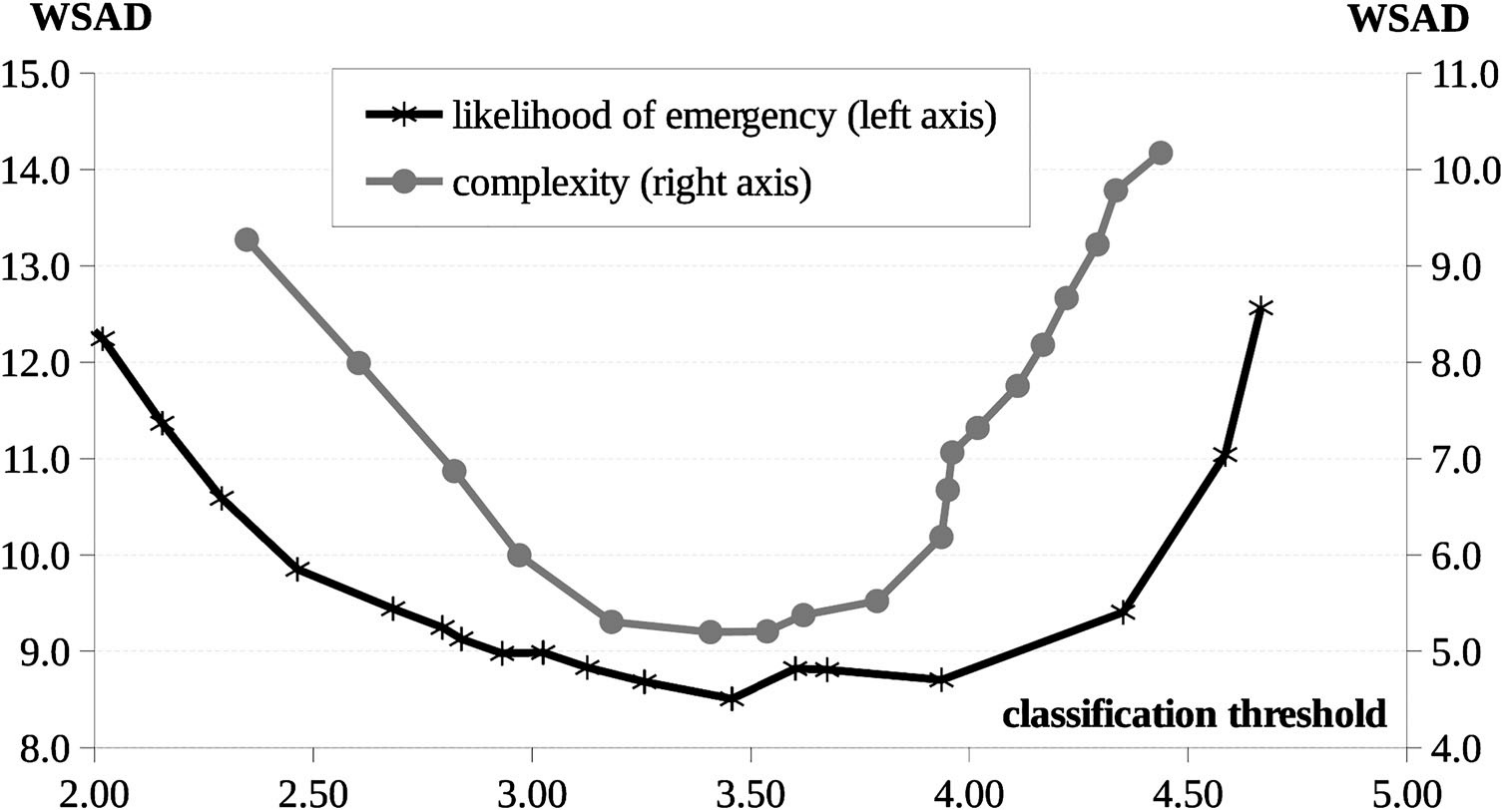
Within-groups sum of absolution deviations (WSAD) for different choices of classification thresholds based on mean ‘complexity’ and ‘likelihood of emergency’ ratings of medical specialties.

Based on the mean ratings we carried out a two-by-two classification of medical specialties. We did this by conducting separately two unidimensional cluster analyses. We arrived at the 2-cluster solution by minimizing the within-groups sum of absolute deviations (WSAD) over the full range of possible cut-values. On the first dimension the WSAD curve had two local minima (see Figure 1). We chose the higher of the two (3.94) for a cut-value, thus classifying the following specialties as ‘of high likelihood of emergency’: surgery, accident surgery, emergency medicine, and intensive care. Choosing the lower cut-value (3.46) would have resulted in classifying three additional specialties as ‘of high likelihood of emergency’: anesthesiology, internal medicine, and obstetrics and gynecology.

On the second dimension we settled with a cut-value of 3.79, classifying the following specialties as ‘highly complex’: internal medicine, anesthesiology, obstetrics and gynecology, neonatology, neurology, psychiatry, general practice, pediatrics, geriatrics, intensive care, and emergency medicine. Here we made a somewhat arbitrary choice, as the WSAD curve flattens out over a range of five consecutive cut-values between 3.18 and 3.79. We chose the highest of these five thresholds, best corresponding with the concept of ‘highly complex’. Choosing the lowest of the five thresholds (3.18) would have resulted in classifying four additional specialties as ‘highly complex’: surgery, accident surgery, oncology, and physiotherapy.

## Results of the survey on intuitive decision-making

Our sample was evenly distributed between physicians (50%) and nurses (50%). The gender composition was markedly different across the two occupations, females making up 56% of physicians and 81% of nurses. As for HCPs’ distribution across medical fields, the most frequent specialties were internal medicine (18.9%), emergency medicine (14.3%), general practice (11.5%), and surgery (10.0%).

Concerning HCPs’ professional experience, our sample exhibited a bimodal distribution, the majority of respondents having either 0–5 years or 20+ years of experience (see Table 2). Female and male physicians’ distribution by work experience was very similar, the difference being non-significant (*χ*^2^(4) = 3.1; *p* = 0.54). In contrast, there was a significant difference between female and male nurses’ distribution (*χ*^2^(4) = 20.3; *p* = 4.4E−04), as female nurses were substantially more experienced than males. We approximated the mean years of experience at 14.9 and 15.2 for female / male physicians, and at 14.4 and 8.0 for female / male nurses.

**Table 2.** Frequency distribution of HCPs by occupation, gender, and professional experience.

Sample counts and row percentages^a^													
Occupation		Years of professional experience											
		0–5 yrs		6–10 yrs		11–15 yrs		16–20 yrs		20+ yrs		Total	
physician		71	31%	24	10%	20	9%	24	10%	91	40%	230	100%
gender	female	43	34%	12	9%	8	6%	13	10%	52	41%	128	56%
	male	28	27%	12	12%	12	12%	11	11%	39	38%	102	44%
nurse		85	37%	26	11%	25	11%	27	12%	67	29%	230	100%
gender	female	56	30%	22	12%	22	12%	25	13%	61	33%	186	81%
	male	29	66%	4	9%	3	7%	2	5%	6	14%	44	19%

^a^column percentages in the ‘Total’ column.

To get a notion about the quality (in terms of representativeness) of the data we had collected, we compared, on the main dimensions, the composition of the sample with that of the population of Hungarian physicians active in 2014 (for nurses such data was not available). We used Hungarian Yearbook of Health Statistics (Vukovich, 2015) data about the joint distribution of physicians by gender, age, and specialty. Years of experience were approximated by subtracting 30 (the typical age for earning a specialist qualification) from the age of the physician; furthermore, we grouped individuals to the three collapsed categories of professional experience.^2^ We also classified physicians as belonging to one of the three groups of medical specialties which we had defined, only taking account of specialties represented in the sample, and discarding physicians (8% of all) in other specialties.

Despite the uncontrolled sampling procedure, we found the main characteristics of the sample to be rather similar to those of the population. The distribution of physicians across medical specialties approximated well the population distribution, the difference in relative frequencies being less than 2%point for almost every specialty (see Table 1). The only remarkable exception was emergency medicine, which was strongly overrepresented.

Overall, the sample was balanced in terms of gender composition, although males were noticeably underrepresented (sample: 44%, pop.: 54%). The main patterns by character of medical specialty were by-and-large well preserved: the majority of physicians were employed in specialties of ‘high complexity’; furthermore, males prevailed in specialties of ‘high likelihood of emergency’, and females prevailed in the two other groups of specialties (see Tables 3 and 4). Having a closer look at the proportions across the three groups of specialties revealed one salient disparity: physicians in specialties of high likelihood of emergency were substantially overrepresented among novices (sample: 25%, pop.: 15%), and even more so among female novices.

**Table 3.** Frequency distribution of physicians by gender, character of medical specialty, and professional experience.

Sample counts and column percentages^a^									
Gender		Level of professional experience							
		novice (0–5 yrs)		advanced (6–15 yrs)		expert (16+ yrs)		Total	
female		43	34%	20	16%	65	51%	128	100%
character of medical specialty	high likelihood of emergency	10	23%	3	15%	6	9%	19	15%
	high complexity	24	56%	14	70%	39	60%	77	60%
	neither	9	21%	3	15%	20	31%	32	25%
male		28	27%	24	24%	50	49%	102	100%
character of medical specialty	high likelihood of emergency	8	29%	8	33%	15	30%	31	30%
	high complexity	15	54%	10	42%	28	56%	53	52%
	neither	5	18%	6	25%	7	14%	18	18%

^a^row percentages in the ‘female’ and ‘male’ rows.

**Table 4.** Distribution of physicians practicing in Hungary by gender, character of medical specialty, and professional experience.

Population counts and column percentages^a^									
Gender		Level of professional experience							
		novice (0–5 yrs)		advanced (6–15 yrs)		expert (16+ yrs)		Total	
female		1701	12%	3689	25%	9323	63%	14713	100%
character of medical specialty	high likelihood of emergency	127	7%	319	9%	492	5%	938	6%
	high complexity	1018	60%	2332	63%	6381	68%	9731	66%
	neither	556	33%	1038	28%	2450	26%	4044	27%
male		1221	7%	3709	21%	12358	71%	17288	100%
character of medical specialty	high likelihood of emergency	239	20%	1126	30%	3279	27%	4644	27%
	high complexity	617	51%	1851	50%	7406	60%	9874	57%
	neither	365	30%	732	20%	1673	14%	2770	16%

^a^row percentages in the ‘female’ and ‘male’ rows.

Professional experience was the only dimension on which the composition of the sample and the population were markedly different, novices being strongly overrepresented (sample: 31%, pop.: 9%) and experts being substantially underrepresented (sample: 50%, pop.: 68%). This could be due to various reasons. Most probably, the on-line survey format combined with chain-referral sampling had as a consequence that younger people were reached in disproportionately greater numbers than older individuals, who tend to rely less on electronic communication. It could also be that the response rate was for some reasons lower among older individuals, hence a possible source of uncontrolled self-selection bias.

### Bivariate analyses

The primary variable of interest was HCPs’ propensity to resort to IDM in their practice. Altogether 185 respondents (40%) admitted ever relying on IDM. This proportion differed significantly across occupations (*χ*^2^(1) = 20.0; *p* = 7.9E−06), physicians being more likely (50%) and nurses being less likely (30%) to rely on intuition. Among physicians as well as among nurses, women exhibited a greater propensity for IDM than men, even though the difference wasn’t statistically significant (physicians: *χ*^2^(1) = 2.1; *p* = 0.15, nurses: *χ*^2^(1) = 0.65; *p* = 0.42).

HCPs’ propensity for IDM was strongly related to their work experience. Overall, the proportion of respondents who reported relying on IDM increased from 25% for novice HCPs to 56% for HCPs with 20+ years of experience. However, this pattern was markedly heterogeneous across occupations (see Table 5). Among physicians a sharp increase could be observed at the transition from 11–15 to 16–20 years of experience. In contrast, the experience-related increase in nurses’ propensity for IDM was much smaller, and it occurred gradually within the first 15 years of their career. For the sake of tractability, in subsequent analyses we collapsed the 5 initial categories of experience to 3 simpler categories: ‘novice’ (0–5 years), ‘advanced’ (6–15 years), and ‘expert’ (16+ years). We believe this simplification is reasonable and the collapsed categories capture the essential pattern of relationship between HCPs’ level of experience and their reliance on IDM.

**Table 5.** Prevalence of intuitive decision-making among HCPs by occupation, gender, and professional experience.

Number and proportion^a^ of HCPs who admitted relying on IDM in their practice													
Occupation		Years of professional experience											
		0–5 yrs		6–10 yrs		11–15 yrs		16–20 yrs		20+ yrs		Total	
physician		20	28%	9	38%	7	35%	15	63%	65	71%	116	50%
gender	female	14	33%	6	50%	3	38%	8	62%	39	75%	70	55%
	male	6	21%	3	25%	4	33%	7	64%	26	67%	46	45%
nurse		19	22%	8	31%	9	36%	10	37%	23	34%	69	30%
gender	female	11	20%	8	36%	9	41%	9	36%	21	34%	58	31%
	male	8	28%	0	0%	0	0%	1	50%	2	33%	11	25%

^a^number who admitted relying on IDM over total count.

To explore how HCP’s propensity for IDM was related to the specialty in which they were practicing, we defined three mutually exclusive groups of medical specialties based on the previously discussed two-way classification (see Table 1). Group 1 comprises four specialties classified as ‘of high likelihood of emergency’ (regardless of level of complexity): surgery, accident surgery, emergency medicine, and intensive care. Group 2 comprises nine specialties classified as ‘of high complexity’ alone (i.e. uncombined with high likelihood of emergency): internal medicine, anesthesiology, obstetrics and gynecology, neonatology, neurology, psychiatry, general practice, pediatrics, and geriatrics. All other specialties, classified as ‘neither highly complex, nor of high likelihood of emergency’, belong to group 3.

Overall 22%, 56%, and 22% of physicians and 37%, 50%, and 13% of nurses practiced in specialties belonging to groups 1, 2, and 3, respectively (see Table 6). For physicians as well as for nurses, character of medical specialty and professional experience could be considered as independent of each other (physicians: *χ*^2^(4) = 1.7; *p* = 0.78, nurses: *χ*^2^(4) = 4.4; *p* = 0.36), i.e. HCPs’ distribution by level of experience didn’t significantly differ across the three groups of specialties.

**Table 6.** Frequency distribution of HCPs by occupation, professional experience, and character of medical specialty.

Sample counts and row percentages^a^									
Occupation		Level of professional experience							
		novice (0–5 yrs)		advanced (6–15 yrs)		expert (16+ yrs)		Total	
physician		71	31%	44	19%	115	50%	230	100%
character of medical specialty	high likelihood of emergency	18	36%	11	22%	21	42%	50	22%
	high complexity	39	30%	24	18%	67	52%	130	56%
	neither	14	28%	9	18%	27	54%	50	22%
nurse		85	37%	51	22%	94	41%	230	100%
character of medical specialty	high likelihood of emergency	37	44%	14	17%	33	39%	84	37%
	high complexity	37	32%	28	24%	50	43%	115	50%
	neither	11	35%	9	29%	11	35%	31	13%

^a^column percentages in the ‘Total’ column.

HCPs’ propensity for IDM was largely influenced by the character of their medical specialty (see Table 7). Considering the pooled sample of physicians and nurses, the proportion of those who reported relying on IDM was substantially greater in specialties ‘of high complexity’ (43%) and ‘of high likelihood of emergency’ (42%) than in specialties classified as ‘neither highly complex, nor of high likelihood of emergency’ (30%). However, this pattern appeared to be stronger for physicians than for nurses, implying a statistically significant difference across the three groups of specialties in the first case (*χ*^2^(2) = 7.2; *p* = 0.028) but not in the second (*χ*^2^(2) = 3.2; *p* = 0.20). It is worthy of notice that the pattern of relationship we had found between HCPs’ professional experience and their reliance on IDM appeared to be universally valid across different types of medical specialties. That is, in all three groups of specialties and for both occupations an overall increasing trend was observable, with an additional sharp increase occurring among physicians at the transition from advanced to expert level.

**Table 7.** Prevalence of intuitive decision-making among HCPs by occupation, professional experience, and character of medical specialty.

Number and proportion^a^ of HCPs who admitted relying on IDM in their practice									
Occupation		Level of professional experience							
		novice (0–5 yrs)		advanced (6–15 yrs)		expert (16+ yrs)		Total	
physician		20	28%	16	36%	80	70%	116	50%
character of medical specialty	high likelihood of emergency	6	33%	4	36%	16	76%	26	52%
	high complexity	13	33%	9	38%	51	76%	73	56%
	neither	1	7%	3	33%	13	48%	17	34%
nurse		19	22%	17	33%	33	35%	69	30%
character of medical specialty	high likelihood of emergency	11	30%	7	50%	13	39%	31	37%
	high complexity	7	19%	8	29%	16	32%	31	27%
	neither	1	9%	2	22%	4	36%	7	23%

^a^number who admitted relying on IDM over total count.

Another focal point of our research was to examine the extent to which IDM was incorporated to HCPs’ education, and whether this had any impact on their reliance on IDM. 45% of all respondents reported that the topic of IDM had never been brought up during their studies or at vocational trainings. 45% reported that they had discussed this topic to a lesser extent, and only 10% had covered it extensively in their formal education. This pattern wasn’t perfectly homogeneous across occupations (*χ*^2^(2) = 9.2; *p* = 0.010), as nurses tended to be somewhat better educated about IDM than physicians (see Table 8). Interestingly, nurses’ extent of being educated in the subject wasn’t significantly related to their years of professional experience (*χ*^2^(4) = 5.8; *p* = 0.22), whereas for physicians we found a marginally significant relationship (*χ*^2^(4) = 9.3; *p* = 0.05). An outstanding characteristic in the latter respect was that among novice physicians none (0%) reported having been extensively educated in the subject.

**Table 8.** Frequency distribution of HCPs by occupation, professional experience, and extent of being educated in the subject of IDM.

Sample counts and column percentages									
Occupation		Level of professional experience							
		novice (0–5 yrs)		advanced (6–15 yrs)		expert (16+ yrs)		Total	
physician		71	100%	44	100%	115	100%	230	100%
extent of being educated in the subject of IDM	not at all	37	52%	19	43%	58	50%	114	50%
	scarcely	34	48%	19	43%	49	43%	102	44%
	extensively	0	0%	6	14%	8	7%	14	6%
nurse		85	100%	51	100%	94	100%	230	100%
extent of being educated in the subject of IDM	not at all	38	45%	15	29%	40	43%	93	40%
	scarcely	33	39%	27	53%	45	48%	105	46%
	extensively	14	16%	9	18%	9	10%	32	14%

Concerning the possibility of HCPs’ propensity for IDM being influenced by whether and to what extent this topic had been discussed in their studies, for nurses we didn’t find any such relationship (*χ*^2^(2) = 3.9; *p* = 0.14). For physicians, in contrast, having been extensively educated in the subject apparently had a large positive impact (*χ*^2^(1) = 6.9; *p* = 8.8E−03), resulting in a 30%points greater proportion of reported IDM usage compared with physicians who had not or only scarcely been exposed to the topic of IDM during their studies (see Table 9). However, this seemingly strong effect might be confounded, as having been extensively educated in the subject of IDM was positively associated with higher levels of professional experience, which in turn had a positive impact on physicians’ reliance on IDM. To disentangle these (and other) direct and indirect effects, we turned to regression analysis.

**Table 9.** Prevalence of intuitive decision-making among HCPs by occupation, professional experience, and extent of being educated in the subject of IDM.

Number and proportion^a^ of HCPs who admitted relying on IDM in their practice									
Occupation		Level of professional experience							
		novice (0–5 yrs)		advanced (6–15 yrs)		expert (16+ yrs)		Total	
physician		20	28%	16	36%	80	70%	116	50%
extent of being educated in the subject of IDM	not at all	11	30%	9	47%	36	62%	56	49%
	scarcely	9	26%	3	16%	37	76%	49	48%
	extensively	0	NA	4	67%	7	88%	11	79%
nurse		19	22%	17	33%	33	35%	69	30%
extent of being educated in the subject of IDM	not at all	6	16%	3	20%	13	33%	22	24%
	scarcely	9	27%	10	37%	19	42%	38	36%
	extensively	4	29%	4	44%	1	11%	9	28%

^a^number who admitted relying on IDM over total count

### Logistic regression analysis

To evaluate the partial effects of all previously mentioned factors on HCPs’ propensity for IDM, we estimated multiple versions of a binary logistic regression model. The variables were as follows:

- [IDM_practice] was the binary response variable, indicating whether the subject admitted relying on IDM in his/her practice;
- [female] was a dummy variable taking a value of 1 for female and 0 for male subjects;
- [physician] was a dummy variable taking a value of 1 for physicians and 0 for nurses;
- [novice] and [expert] were dummy variables indicating whether, regarding his/her years of professional experience, a HCP was classified either as ‘novice’ (0–5 yrs) or as ‘expert’ (16+ yrs);
- [IDM_study_1] and [IDM_study_2] were dummy variables indicating whether and to what extent the topic of IDM had been discussed (_1 for scarcely, _2 for extensively) during the subject’s medical studies and/or vocational trainings;
- [hgh_compl], [hgh_emerg] were binary variables identifying subjects in specialties classified as ‘highly complex’ or ‘of high likelihood of emergency’.

In some model versions we included dummies coding complementary or merged categories for the variables above, i.e. [male] = 1–[female], [nurse] = 1–[physician], [advanced] = 1–[novice]–[expert], [IDM_study_1p] = [IDM_study_1] + [IDM_study_2]. Additionally, in a maximal model version we included further dummy variables to identify subjects in each of the 20 medical specialties.

To explore in depth the influence of person- and job-specific factors on HCPs’ propensity for IDM, besides estimating the repressors’ main effects we also allowed and tested for a large set of meaningful interaction effects. Thus we carried out a systematic model selection procedure whereby we estimated, compared, and tested for the difference between more than 60 model version, keeping an eye on the robustness of the repressors’ partial effects. In doing so, our objective was to develop a tractable model structure capturing the fundamental patterns of relationship in the data while avoiding overfitting the model.

### Model selection

As the result of model selection we established the following set of relationships between the variables. (1) Gender had a significant main effect on the dependent variable (IDM_practice), without interacting with occupation. (2) Character of medical specialty (high_emerg, high_compl) had a significant main effect as well as an interaction effect with occupation. (3) Professional experience (novice, expert) had a significant main effect and also an interaction effect with occupation, but (as for the latter) only concerning expert level HCPs. In particular, the partial effect of [expert] was large and positive for physicians, whereas for nurses it wasn’t significantly different from zero. (4) Education in the subject of IDM had a non-significant main effect, whereas some (weak) evidence was found for its positive effect in interaction with occupation. In particular, for nurses the non-zero level (IDM_study_1p) and for physicians the highest level (IDM_study_2) of the variable had sizable regression coefficients, which were marginally significant (*p* < 0.10) in some model versions. However, the evidence was altogether inconclusive.

We’ll present and discuss our final model structure (2) in comparison with two other models (1, 3). In all three cases we considered two alternative model versions (a, b), differing as to whether interaction terms between the levels of [IDM_study] and occupation were included or not (see Table 10). Since we didn’t find conclusive evidence for the non-zero effect of these additional terms, they were only added as control variables for the purpose of robustness check.

**Table 10.** Factors of intuitive decision-making and their estimated regression coefficients in different versions of a binary logistic regression model.

	Model 1/a		Model 1/b		Model 2/a		Model 2/b		Model 3/a		Model 3/b	
const	−0.86	***	−1.01	***	−1.73	***	−1.87	***	−20.41		−20.69	
female	0.29		0.28		0.52	**	0.50	**	0.50	*	0.47	*
novice	−0.43	*	−0.34		−0.54	**	−0.46	*	−0.59	**	−0.50	*
expert × phys	1.52	***	1.61	***	1.41	***	1.48	***	1.50	***	1.56	***
hgh_emerg × phys					1.24	***	1.25	***				
hgh_compl × phys					1.15	***	1.17	***				
hgh_emerg × nurse					1.07	***	0.91	**				
hgh_compl × nurse					0.41		0.31					
med_spec_1-20 × phys									(…)		(…)	
med_spec_1-20 × nurse									(…)		(…)	
IDM_study_1p × nurse			0.24				0.41				0.47	
IDM_study_2 × phys			1.41	**			1.19	*			1.15	
McFadden *R*-squared	0.097		0.106		0.126		0.134		0.174		0.182	
Log-likelihood	−279.9		−277.2		−271.1		−268.6		−256.0		−253.7	

*p < 0.1, **p < 0.05, ***p < 0.01.

### Validating the dimensions of complexity and likelihood of emergency

The reason for presenting models 1 and 3 along with model 2 is to assess how well our proposed two-way classification (as represented by the dummies [high_emerg] and [high_compl]) captures the variability in HCPs’ propensity for IDM across medical specialties. Model 3 is an enhanced, maximal variant of model 2, in which individual dummies are included (in interaction with occupation) for each of the 20 specialties, thus taking full account of their particularities as to how much they call for and accommodate IDM. Model 1, on the other hand, is a reduced variant of model 2, taking no account of the effect of medical specialty at all. Comparing the log-likelihood values of the three models reveals that our proposed two-way classification accounts for 37% of the total explanatory power of the 20 specialties. This is a substantial proportion, especially taking into account that model 3 severely overfits the data, so it should only be considered as a hypothetical benchmark for evaluation.

Comparing the regression coefficients across models 2 and 3 provides further validation for the two-way classification which we used in our final model (2). The robustness of estimated coefficients suggests that representing medical specialties in this simplified way, with two dummies only, doesn’t produce any confounding effect as to how HCPs’ reliance on IDM is influenced by other person- and job-specific factors.

### Determinants of intuitive decision-making and their partial effects

We used model version (2/a) to predict the dependent variable (IDM_practice) and evaluate the partial effect of the repressors. As a starting point we calculated, for any combination of subject characteristics, the estimated probability of a HCP relying on IDM in his/her practice (see Table 11).

**Table 11.** HCP’s propensity for intuitive decision-making by occupation, gender, professional experience, and character of medical specialty, as estimated within a binary logistic regression model (version 2/a).

Estimated conditional probabilities^a^ of relying on IDM							
Level of professional experience		Occupation					
		physician	gender		nurse	gender	
			female	male		female	male
novice (0-5 yrs)		28%	32%	22%	22%	23%	20%
character of medical specialty	high likelihood of emerg.	33%	38%	26%	28%	34%	23%
	high complexity	31%	35%	25%	19%	21%	13%
	neither	13%	15%	9%	14%	15%	9%
advanced (6-15 yrs)		38%	45%	31%	33%	35%	20%
character of medical specialty	high likelihood of emerg.	41%	51%	38%	46%	47%	34%
	high complexity	43%	49%	36%	30%	31%	21%
	neither	18%	23%	15%	20%	23%	15%
expert (16+ yrs)		70%	72%	66%	35%	35%	34%
character of medical specialty	high likelihood of emerg.	74%	81%	72%	44%	47%	34%
	high complexity	75%	79%	70%	31%	31%	21%
	neither	52%	55%	42%	23%	23%	15%

^a^Pooled probabilities (across genders and medical specialties) are computed as sample-weighted averages of conditional probabilities.

The model had relatively small explanatory power (McFadden-*R*^2^ = 0.126), indicating that most of the variability in HCPs’ propensity for IDM was attributable to unobserved factors (such as the person’s cognitive style, his/her attitude to IDM, etc.). Nonetheless, the repressors were jointly significant (*χ*^2^(7) = 77.8; *p* = 3.8E−14). Also, the model reproduced rather well the observed pattern of variation in HCPs’ reliance on IDM in relation to their occupation, professional experience, and character of medical specialty (see Table 7), with an average prediction error of 2.8%points (sample-weighted average across the 2 × 3 × 3 joint categories).

Next we computed (in additive as well as in multiplicative form) the repressors’ partial effects on HCPs’ estimated probability of resorting to IDM in their practice. These partial effects were evaluated at the sample mean value of all other repressors, and conditionally on the relevant category in the case of variables for which we had found a significant interaction effect.

As regards effect sizes, we found occupation, professional experience, and field of medicine to be equally strong determinants of HCPs’ propensity for IDM (see Table 12). Physicians’ / nurses’ estimated probability of relying on IDM was, all other things being equal, 0.25/0.20 greater (respectively) in specialties ‘of high likelihood of emergency’ than in specialties classified as ‘other’. The partial effect of practicing in a ‘highly complex’ specialty was of similar magnitude, 0.23 for physicians, whereas for nurses it was much smaller (0.07) and statistically non-significant. (Nonetheless, to keep the model structure coherent we preserved the interaction term [hgh_compl × nurse] in the final model version.) The effect of medical specialty was altogether significant (*χ*^2^(4) = 17.6; *p* = 1.5E−03).

**Table 12.** Factors of intuitive decision-making and their partial effects as calculated within a binary logistic

Variable of interest	Basis of comparison	Conditioning category	Partial effect on logit	2 tailed *p*-value	Partial effect on Pr(IDM=1)	
					additive	multiplicative
female	male	all HCPs	0.524	0.034	0.101	1.31
hgh_emerg	‘other’ specialties	physicians	1.239	3.6E−03	0.247	1.78
		nurses	1.068	5.9E−03	0.204	2.14
		average	1.154	1.1E−03	0.226	1.91
hgh_compl	‘other’ specialties	physicians	1.146	7.7E−04	0.228	1.72
		nurses	0.413	0.273	0.068	1.38
		average	0.779	0.015	0.148	1.59
novice	advanced	all HCPs	−0.542	0.032	−0.108	0.70
expert	advanced	physicians	1.410	9.0E−06	0.314	1.77
physician	nurse	specialties with high likelihood of emergency	0.811	0.040	0.181	1.47
		specialties of high complexity	1.374	3.6E−06	0.299	2.21
		other specialties	0.641	9.0E−06	0.138	1.77
		average	1.081	1.1E−07	0.237	1.86
physician	nurse	novice HCPs	0.440	0.053	0.082	1.39
		advanced HCPs	0.440	0.053	0.100	1.32
		expert HCPs	1.850	1.7E−10	0.414	2.34
		average	1.081	1.1E−07	0.237	1.86

We evaluated the effect of professional experience in comparison with advanced level (6–15 yrs), considered as the reference category. The estimated probability of IDM was on average 0.11 less for novice than for advanced level HCPs, with no significant difference in effect size across physicians and nurses. More interestingly, among nurses there wasn’t any significant difference between advanced and expert level HCPs’ probability of IDM, whereas for physicians the partial effect of being expert was large (0.31) and significant. The effect of experience was altogether highly significant (*χ*^2^(2) = 42.4; *p* = 6.3E−10).

Occupation had a substantial main effect in the model: the estimated probability of relying on IDM was on average 0.24 greater for physicians than for nurses. This effect was largely heterogeneous across medical fields: 0.18 in specialties ‘of high likelihood of emergency’, 0.30 in ‘highly complex’ specialties, and 0.14 in ‘other’ specialties. The effect also varied with professional experience: for novice / advanced / expert physicians the estimated probability of IDM was 0.08/0.10/0.41 greater (respectively) than for nurses with the same level of experience. The effect of occupation was altogether highly significant (main effect: *z* = 5.31; *p* = 1.1E−07, total effect: *χ*^2^(3) = 38.5; *p* = 2.2E−08).

The coefficient of [female] was significant in the regression (*z* = 2.12, *p* = 0.034), providing evidence for gender by itself influencing HCPs’ propensity for IDM. The estimated probability of IDM was, all other things being equal, on average 0.10 greater for women than for men.

Education in the subject of IDM had a non-significant main effect, whereas we found some (weak) evidence for its positive effect in interaction with occupation. In particular, for nurses the non-zero level (IDM_study_1p) and for physicians the highest level (IDM_study_2) of the variable had sizable regression coefficients, which were marginally significant (*p* < 0.10) in some model versions. However, the evidence was altogether inconclusive.

Levels of [IDM_study] aren’t included in model 2/a, and correspondingly their partial effects are not reported in Table 12, as we didn’t find conclusive evidence for HCPs’ propensity for IDM being influenced by the extent of having been educated in the subject. Yet, for the sake of completeness we have considered model output 2/b, which contains positive coefficients for particular levels of [IDM_study] in interaction with occupation (see Table 10). This modified model structure would imply that for physicians the highest level of the variable (having extensively studied the subject of IDM) largely (by 0.24) increases the probability of IDM, whereas its medium level by itself has no effect. For nurses, in contrast, already the medium level of the variable increases the probability of IDM to some extent (by 0.07), whereas switching from medium to high level has no further positive effect. Upper-tail tests about the two partial effects being positive (IDM_study_2 × phys: *z* = 1.65; *p* = 0.050, IDM_study_1p × nurse: *z* = 1.38; *p* = 0.083) provide some support for our research hypothesis (3), but altogether the evidence is insufficient.

## Results of the analysis of free-text case descriptions

To investigate how survey participants understood the concept of ‘intuition’, we took a closer look at their free-text accounts of IDM instances. Out of the 229 case descriptions submitted by 667 HCPs in the unrestricted sample, we identified 140 as containing important characteristics of intuitive information-processing and/or intuitive decision-making. This indicates that, although there was substantial heterogeneity in respondents’ understanding of the term ‘intuition’, and clearly some of the accounts were far from the scientific approach (referring to God or the Universe as the sources of intuition, or mistaking IDM for being influenced by personal likes and dislikes in one’s decisions), the large majority of accounts were fundamentally compatible with our concept of IDM as discussed in the introduction. To exemplify this, we report the literal translation of excerpts from two respondents’ free-text answers.

1. Female nurse, 11–15 years of work experience, psychiatry: *In my view, intuitive decisions emerge when pieces of information picked up at different times and stored in different places in our memory become non-consciously connected in a new and unexpected way, which we reflect upon and embody. Hence I would say that healing professions have an artistic aspect, because such intuitive judgments can only be born out of a deep attunement to the patient, yielding solutions customized to the patient’s needs. Yet, this requires complete devotion from the healer. This way of treatment isn’t something to be learned; it requires one to turn to the patient with all the resources of one’s personality.*
2. Male physician, 20+ years of work experience, pediatrics: *Intuition is a hunch which we have on the basis of our accumulated life experiences but which we cannot clearly articulate. (…) Intuition works properly when we turn all our attention, all our ‘antennas’ to the patient and the circumstances, observing all the tiniest details (such as casual remarks made by people in the patient’s environment). (…) Of course in my work I rely on clinical guidelines in the first place, but I also pay much attention to my intuitions. They can be particularly helpful in rare, unusual situations.*

Next, we performed content analysis on the 140 case descriptions judged as relevant to the topic of IDM. We searched for typical and classifiable elements of these instances, which were either explicit or which could be reasonably inferred from the accounts. We classified such typical elements as belonging to one of four categories: (1) characteristic behavior, (2) modes of cognition, (3) abilities and personal dispositions, and (4) guiding considerations for the decision.

Finally, we grouped the accounts by the respondent’s gender (69% female, 31% male), occupation (55% physician, 20% nurse, 25% other HCP), character of medical specialty (28% high likelihood of emergency, 54% high complexity, 18% neither), level of professional experience (22% novice, 16% advanced, 62% expert), and education received in the subject of IDM (48% none, 52% scarce or extensive), and we calculated within each group the relative frequency of accounts containing each particular element in the four categories as above (see Tables 13–16).

**Table 13.** Characteristic behavior in IDM instances, by gender, occupation, character of medical specialty, professional experience, and extent of being educated in the subject of IDM.

Relative frequency of accounts involving distinctive behaviors													
Behavior	Gender		Occupation		Character of medical specialty			Level of prof. experience			Education about IDM		Total
	fem	male	phys	nurse	h.emrg	h.cmpl	neither	nov	adv	exp	no	yes	
^1)^ Further exam. or treatment	34%	47%	43%	32%	39%	42%	25%	25%	50%	40%	28%	47%	38%
^2)^ Deviation from protocol	25%	32%	23%	32%	26%	24%	40%	33%	22%	26%	32%	23%	27%
^3)^ Intuitive diagnosis	8%	15%	13%	9%	3%	15%	5%	8%	11%	10%	15%	5%	10%
^4)^ Extra vigilance	9%	6%	5%	14%	19%	3%	5%	4%	11%	9%	8%	9%	8%
^5)^ Rapid decision about treatment	4%	15%	7%	14%	16%	5%	0%	8%	6%	7%	9%	5%	7%

^1^Performing or requesting further medical investigation despite lack of symptoms and/or negative examination results;

Applying or requesting additional treatment despite negative or inconclusive indications about its necessity;

Forwarding the patient to hospital care despite negative or inconclusive examination results

^2^Deviating from diagnostic or treatment protocols

^3^Establishing the diagnosis intuitively, despite negative or inconclusive examination results

^4^Monitoring the patient with exceptional vigilance;

Acting on a strong urge to check up the patient’s condition

^5^Making rapid, intuitive treatment decisions despite incomplete or inconclusive examination results;

Making rapid, intuitive treatment decisions despite lack of authorization by supervising physician;

Taking instantaneous, intuitive, common-sense action to deal with emergency or immediate danger

**Table 14.** Modes of cognition involved in IDM instances, by gender, occupation, character of medical specialty, professional experience, and extent of being educated in the subject of IDM.

Relative frequency of accounts involving distinctive modes of cognition													
Mode of cognition	Gender		Occupation		Character of medical specialty			Level of prof. experience			Education about IDM		Total
	fem	male	phys	nurse	h.emrg	h.cmpl	neither	nov	adv	exp	no	yes	
^1)^ Pattern recognition	58%	76%	75%	59%	68%	68%	45%	50%	67%	68%	66%	61%	64%
^2)^ Presentiment	39%	38%	42%	45%	45%	44%	15%	46%	33%	38%	36%	42%	39%
^3)^ Insights from attunement to pt.	33%	18%	22%	32%	23%	31%	30%	33%	33%	25%	26%	30%	28%
^4)^ Metacognition	8%	9%	12%	5%	6%	10%	5%	4%	17%	7%	4%	12%	8%

^1^Non-conscious / automatic pattern recognition

^2^Presentiment; use of affect as information

^3^Gaining intuitive insights through emotional attunement to the patient

^4^Metacognition; consciously reflecting on one’s mental processes

**Table 15.** Abilities and personal dispositions involved in IDM instances, by gender, occupation, character of medical specialty, professional experience, and extent of being educated in the subject of IDM.

Relative frequency of accounts implying distinctive abilities and personal dispositions													
Ability or personal disposition	Gender		Occupation		Character of medical specialty			Level of prof. experience			Education about IDM		Total
	fem	male	phys	nurse	h.emrg	h.cmpl	neither	nov	adv	exp	no	yes	
^1)^ See the patient as a whole	55%	35%	48%	50%	48%	51%	45%	54%	67%	43%	45%	53%	49%
^2)^ Attentiveness to subtle signs	7%	18%	12%	0%	10%	8%	15%	4%	17%	10%	13%	7%	10%
^3)^ Strong reliance on experience	25%	47%	30%	36%	23%	36%	35%	21%	33%	35%	34%	30%	32%
^4)^ Sense of reassurance	28%	21%	25%	32%	29%	19%	40%	25%	22%	26%	23%	28%	25%
^5)^ Sense of alarm	24%	24%	27%	14%	26%	25%	15%	29%	28%	21%	19%	28%	24%
^6)^ Empathy & emot. attunement	21%	9%	10%	27%	13%	17%	25%	13%	28%	16%	15%	19%	17%
^7)^ Responsibility	21%	15%	17%	32%	32%	17%	5%	33%	17%	15%	17%	21%	19%
^8)^ Thoroughness	18%	12%	18%	9%	23%	15%	10%	8%	17%	19%	11%	21%	16%
^9)^ Proactivity	7%	18%	7%	23%	23%	7%	0%	21%	17%	4%	13%	7%	10%
^10)^ Courage	4%	15%	8%	9%	6%	8%	5%	8%	0%	9%	9%	5%	7%
^11)^ Receptiveness to new info	4%	9%	7%	5%	10%	3%	5%	4%	11%	4%	8%	4%	5%

^1^Attentiveness to the patient’s overall perceptual and behavioral aspects

^2^Attentiveness to subtle signs indicative about the patient’s clinical condition

^3^Strong reliance on experience-based knowledge

^4^Receptiveness to intuitive insights (sense of reassurance, sense of ‘everything fits in’);

Trusting one’s intuitive insights

^5^Receptiveness to sinister presentiments (sense of alarm, sense of ‘something doesn’t fit in’);

Using presentiments as signals of need for vigilance

^6^Disposition for empathy and emotional attunement to the patient

^7^Strong sense of responsibility; dedication to helping and relieving patients

^8^Thoroughness; dedication to attaining an optimal diagnostic / treatment result

^9^Proactivity; readiness to autonomously take rapid and vigorous action

^10^Courage and sense of competence required to deviate from the protocol

^11^Readiness to acquire and integrate new information; willingness to revise first impressions

**Table 16.** Guiding considerations mentioned in relation to IDM instances, by gender, occupation, character of medical specialty, professional experience, and extent of being educated in the subject of IDM.

Relative frequency of accounts mentioning distinctive considerations													
Guiding consideration	Gender		Occupation		Character of medical specialty			Level of prof. experience			Education about IDM		Total
	fem	male	phys	nurse	h.emrg	h.cmpl	neither	nov	adv	exp	no	yes	
^1)^Use info from multiple sources	32%	29%	35%	27%	29%	32%	30%	25%	39%	31%	21%	40%	31%
^2)^Prevent harms	9%	21%	15%	5%	19%	10%	10%	21%	6%	12%	15%	11%	13%

^1^Reliance on different kinds of diagnostic and prognostic information from multiple sources

^2^Prevention of fatal / irreparable outcome;

Prevention of harms caused by insufficient or delayed treatment;

Prevention of harms caused by sticking to the protocol

The most frequently (38%) mentioned behavior involved in IDM instances was performing or requesting further medical investigation and/or additional treatment (including hospital care) despite negative or inconclusive examination results (1). In second place, some kind of deviation from the medical protocol (2) was mentioned in 27% of cases. Less frequent behaviors included establishing the diagnosis in an intuitive way (3; freq. = 10%), acting on a strong urge to check up the patient’s condition or exercising some other form of exceptional vigilance (4; freq. = 8%), and making rapid, intuitive treatment decisions in response to emergencies (5; freq. = 7%). Interestingly, male HCPs appeared to be generally more inclined to engage in (or more willing to admit) these behaviors than females.

We found a remarkable complementarity between the two most widely observed behaviors as for how they were affected by HCPs’ individual characteristics. Performing or requesting further medical examination or treatment (1) appeared more frequently in the accounts of physicians, HCPs employed in specialties of high complexity and/or high likelihood of emergency, HCPs with higher levels of experience, and HCPs who had received some extent of formal education in the subject of IDM. In contrast, deviating from the protocol (2) was mentioned more often by nurses, by HCPs in specialties of relatively low complexity and low likelihood of emergency, by novice HCPs, and by HCPs who hadn’t been educated in the subject of IDM. As for the three less common behaviors, establishing an intuitive diagnosis (3) seemed to occur most often in specialties of high complexity as well as among HCPs uneducated in the subject of IDM, whereas exercising extra vigilance over the patient (4) and rapidly engaging in the provision of medical treatment (5) was reported most often by nurses and by HCPs in specialties of high likelihood of emergency.

Concerning the characteristic modes of cognition, non-conscious / automatic pattern recognition (1) was identifiable in the large majority (64%) of accounts. Males seemed more likely than females, and physicians seemed more likely than nurses to rely on this mode of cognition, and also it was reported more often by HCPs in specialties of high complexity and/or high likelihood of emergency, as well as by HCPs with higher levels of experience. Presentiments and the use of affect as information (2) appeared in 39% of accounts, males and females, physicians and nurses being equally likely to mention it. Yet, it seemed to occur more frequently in specialties of high complexity and/or high likelihood of emergency, as well as among novice HCPs. Gaining intuitive insights through emotional attunement to the patient (3) was mentioned in altogether 28% of cases, substantially more frequently by females than by males, and also more frequently by nurses than by physicians. In a minor proportion (8%) of accounts we found evidence for HCPs engaging in metacognition (4), i.e. consciously reflecting on their mental processes during the IDM instance. This mode of cognition was reported in higher proportion by physicians and by HCPs educated in the subject of IDM.

Concerning the abilities and the personal dispositions involved in IDM instances, attentiveness to the patient’s overall perceptual and behavioral aspects (1) was mentioned (or implied) in 49% of accounts, females being apparently more likely than males to exhibit this disposition. As an interesting contrast, attentiveness to subtle signs indicative about the patient’s clinical condition (2; freq. = 10%) was mentioned more frequently in males’ than in females’ accounts. These results indicate a possible difference between males’ and females’ ways of perception, men being more disposed to focus on smaller details, whereas women being more perceptive to the ‘big picture’.

Another salient characteristic, strong reliance on experience-based knowledge (3; freq. = 32%) was mentioned predominantly by males, and most often by HCPs with higher levels of experience. Receptiveness to one’s intuitive insights (sense of reassurance; 4) and receptiveness to sinister presentiments (sense of alarm; 5) also seemed to play an important role in instances of IDM; these dispositions were mentioned (or implied) in 25% and 24% of accounts, respectively. We suggest there might be some degree of complementarity between these two dispositions, as sense of alarm (5) was reported more often by physicians and by HCPs in specialties of high complexity and/or high likelihood of emergency, whereas sense of reassurance (4) appeared more frequently in the accounts of nurses and HCPs in specialties of relatively low complexity and low likelihood of emergency.

Disposition for empathy and emotional attunement to the patient (6; freq. = 17%) was found predominantly in females’ and in nurses’ accounts. Strong sense of responsibility (7) and thoroughness in one’s work (8) also seemed to be involved in HCPs’ instances of IDM; evidence for these qualities was found in 19% and 16% of cases, respectively. Proactivity (9) and courage (10) appeared in a smaller proportion (10% and 7%) of accounts, typically in situations calling for rapid, vigorous action and/or deviation from the protocol; also, they were mentioned much more often by male than by female HCPs. We found an interesting pattern concerning proactivity (9) and sense of responsibility (7): both dispositions were strongly positively associated with being a nurse, being less experienced (probably in relation to start-of-career enthusiasm), and working in a specialty of high likelihood of emergency. Readiness to acquire and integrate new information (11), an important cognitive disposition, was mentioned (or implied) only in a minor fraction (5%) of accounts. Interestingly, none of these eleven abilities and dispositions seemed to be significantly related to whether the individual had received formal education in the subject of IDM.

Lastly, we identified two salient factors which HCPs had mentioned as guiding considerations for their decisions, either in the specific IDM instance described, or in situations calling for IDM in general. The foremost such consideration was relying on and trying to integrate multiple pieces of diagnostic and prognostic information (1) including current symptoms, patient history, general perceptual impressions about the patient and her/his environment, physiologic and pathologic measurements, illness scripts, illness presentations previously encountered, and general biomedical facts. This consideration was mentioned (or implied) in 31% of all accounts, this proportion being practically independent of respondents’ gender, occupation, specialty, professional experience, and also unrelated to the particular behavior(s) involved in the IDM instance. In contrast, it was strongly positively related to respondents’ educational background: those who had received some education in the subject of IDM were 2 times more likely to mention this consideration than those who hadn’t.

The other guiding consideration was the prevention of harms to the patient (especially, a fatal or irreparable outcome) caused by insufficient or delayed treatment (2). Overall, this consideration appeared in a smaller fraction (13%) of accounts, and it was mentioned mainly by males, by physicians, by novice HCPs, and by HCPs working in specialties of high likelihood of emergency. Also, it seemed to occur with substantially greater frequency (24%) in IDM instances involving further medical examination and/or additional treatment of the patient despite negative or inconclusive examination results.

## Discussion

We have argued that intuitive and other non-analytical ways of DM are essential in medical practice, due to the inherent uncertainty, incomplete information, and time pressure, which are characteristic of the circumstances of CDM. Indeed, 40% of HCPs in our sample admitted relying on intuition in their decisions, providing dozens of detailed accounts of instances of IDM.

Before going any further with the discussion, an important issue concerning the internal validity of our results is to what extent positive/negative answers to the main survey question are indicative about HCPs’ true propensity for IDM. It is reasonable to consider the proportion of positive answers as a lower bound on the true proportion of participants who do resort to IDM in their practice. Indeed, it is difficult to think of any situation in which a nurse or a physician should claim that she/he occasionally makes diagnostic or treatment decisions in an intuitive way whereas in fact she/he doesn’t. The opposite situation, however, is much more likely to arise.

Continuing this line of thought, it makes sense to assume that a positive answer to the main survey question was dependent on three conditions: (1) the person does in fact resort to IDM; (2) she/he is aware of this fact; (3) she/he is willing to admit this fact. So the total variability in HCP’s true propensity for IDM could in theory be broken down to these three factors, all of which are possibly affected by HCPs’ individual and job-related characteristics. E.g. the positive relationship we have found between physicians’ professional experience and their propensity for positively answering the main survey question can be the result of (1) more experienced physicians relying on IDM to a greater extent; (2) such physicians having greater self-reflective ability and thus being more aware of their cognitive processes during DM; (3) such physicians being professionally better established and more confident, thus being less reluctant to admit their reliance on IDM; or any combination of the former three. Within the framework of our research there was no way of disentangling these different mechanisms, so all one can do is to bear them in mind when interpreting the results.

Next, our objective was to identify the relevant factors influencing HCPs’ propensity for IDM and see how well our results, obtained in a medical context, fit with prior findings about the circumstances calling for and the individual characteristics facilitating IDM. Four out of the five research hypothesis we had formulated on these grounds were confirmed by the statistical analysis, whereas in one case the evidence was insufficient.

### Job-specific factors of intuitive decision-making

It makes sense to categorize the factors influencing HCPs’ reliance on intuition as either person- or job-specific; gender, educational background, and professional experience belonging to the former, whereas occupation and field of medicine belonging to the latter category. Concerning the role of field of medicine, prior research focused mostly on the particularities of CDM within specific fields. Considerable interest in this matter has been devoted to emergency medicine, a specialty characterized by extreme degrees of time pressure, unpredictability, and complexity of medical scenarios (Zavala et al., 2018). It has been found that, because of its singular circumstances, normative DM procedures are in many cases impracticable in emergency medicine, and alternative (intuitive, heuristic) ways of CDM are used extensively, instead (Coget & Keller, 2010).

In this respect, our research is unique for investigating the role of complexity and likelihood of emergency separately and across a variety of specialties. We have found that both aspects, independently of each other, increase clinicians’ reliance on IDM. This suggests that the need for intuitive methods in CDM is related to two distinctive aspects of medical scenarios: (1) the necessity of making instantaneous decisions in unforeseeable situations; (2) the necessity of considering multiple perceptual and diagnostic aspects of patients’ condition along with a variety of treatment options and their possible effects. These results are consistent with research findings on human DM in other areas showing that time pressure, unpredictability, and complexity are prominent characteristics of situations in which IDM is most adaptive (Agor, 1986; Hogarth, 2005; Okoli & Watt, 2018).

As for the role of occupation, our results point in the direction that higher responsibility jobs (physician) call for IDM to a greater extent than lower responsibility jobs (nurse). This is congruent with findings about people in high-responsibility positions being more disposed to adopt experience-based, non-analytical ways of DM (Agor, 1986; Klein, 1999). Results along the same line in a medical context were obtained by Price, Zulkosky, White, and Pretz (2017).

### Person-specific factors of intuitive decision-making

Among the person-specific factors we have found professional experience to have a large positive impact on HCPs’ propensity for IDM. This is in line with research showing that expert clinicians tend to rely on IDM more extensively than non-experts (Benner & Tanner, 1987; Miller & Hill, 2018; Pretz & Folse, 2011). Non-analytic ways of information processing which become available through clinical expertise have also been suggested to contribute to diagnostic efficiency at multiple stages of the CR process (King & Clark, 2002; Schmidt & Rikers, 2007; Van De Wiel, Boshuizen, & Schmidt, 2000). In possession of a large experiential knowledge base, expert clinicians are able to generate valid diagnostic hypotheses through an automatic, non-conscious pattern-matching process (Brush et al., 2017; Norman et al., 2007), and often they come to a conclusive diagnosis very quickly, by making efficient cognitive shortcuts in the analytical test procedure (Groen & Patel, 1985). These cognitive pathways are congruent with the positive relation we have found between clinical expertise and IDM.

Interestingly, for physicians the relationship we have found between level of expertise and reliance on IDM is markedly non-linear, a sharp increase occurring at the transition from 11–15 to 16–20 years of experience. This suggests that clinicians at a certain stage of proficiency undergo a substantial change in their information processing. This is congruent with Dreyfus and Dreyfus (1986) model of skill acquisition, whereby switching from analytical to intuitive DM is a hallmark of the transition from ‘proficient’ to ‘expert’ level.

Building on Schema Theory (e.g. Rumelhart, 1980) and research about the cognitive background of expert performance, Mero (2002) has argued that reaching this highest level of expertise requires the accumulation of tens of thousands of cognitive schemata specific to the field of knowledge, entailing a degree of cognitive complexity beyond the limits of analytic thinking. Research about clinicians’ knowledge structure has identified different types of such schemata, from symbolically represented biomedical facts and illness scripts to an implicit, experience-based network of diagnostic categories comprising innumerable instances of symptom presentations (Brush et al., 2017; Norman & Brooks, 1997; Schmidt & Rikers, 2007). As clinicians gain expertise, their repertory of experientially acquired cognitive schemata expands, and their CR processes become more reliant on tacit knowing (Brush et al., 2017; Van De Wiel et al., 2000). This corresponds well with our findings about expert physicians’ propensity for IDM increasing by a large factor.

As an intriguing result, for nurses we have found no evidence of such a major shift from analytical to non-analytical CDM. In comparison with physicians, nurses’ propensity for IDM increased to a lesser extent, much more gradually, and relatively early in their career. This raises the possibility that nurses’ skill acquisition and/or the development of their knowledge structure follows a different path than that of physicians. Investigating this issue would require further research.

We have found gender as a second person-specific factor of HCPs’ reliance on intuitive judgment, female HCPs being more likely to resort to IDM than males. Although this finding is in line with conventional wisdom about women being generally more intuitive than men, there is as yet scarce evidence for such phenomena in a clinical context. One exception is a small sample study by Woolley and Kostopoulou (2013), who have found that female family physicians are more receptive to gut feelings in their diagnostic work than males. Nonetheless, further research would be necessary to explore the cognitive and personality factors underlying any difference between male and female clinicians’ preferred ways of CDM. (We have partially undertaken this in the current study by way of systematic content analysis of respondents’ free-text accounts of IDM instances.)

Besides gender and professional experience, we have found some (weak) evidence for a third person-specific factor of HCPs’ propensity for IDM: the extent to which a person has been educated about the use of intuition in CDM. For physicians the highest level of this factor [IDM_study_2] had a considerable positive effect, yet the data was insufficient to confirm its existence at the population level. This was a consequence of the small proportion (6%) of physicians in this category, reflecting the fact that the topic of IDM hasn’t been effectively incorporated to the curricula of medical study programs in Hungary.

Many have argued that the development of CR skills – including intuitive thinking – should be as much part of the training of medical students as teaching them the core body of biomedical knowledge (Bowen, 2006; Marcum, 2012; Pelaccia et al., 2011). This would entail (1) encouraging students to freely express their intuitive thoughts about medical cases, (2) teaching them to self-reflect on what clinical and contextual cues they have used for forming an intuitive impression, and (3) giving them instantaneous feedback about the diagnostic hypotheses and conclusions they have come to. Furthermore, medical and nursing students should be exposed to a large number of clinical cases so that they start to build up a repertory of symptom patterns, serving as basis for their intuitive CR skills (Eva, 2005; Marcum, 2012; Schmidt & Rikers, 2007). These arguments, in line with our empirical findings, support the claim that the prevalence and the quality of clinicians’ IDM could be enhanced by way of teaching; even though the current practice of medical education is far from having reached its full potential in this respect.

### Behavioral and cognitive aspects of intuitive decision-making

Besides exploring the person- and job-specific factors of HCPs’ reliance on IDM, our research provided insight to the nature, the circumstances, the cognitive background, and the beneficial use of intuitive decisions in clinical practice. Such insights were obtained from personal accounts of instances of IDM given by HCPs who had participated in our first survey. Based on these accounts we identified several core attributes which appeared, to varying degrees, characteristic of clinicians’ cognition and behavior in such instances, and we investigated how these were related to HCPs’ job-related and individual features. A few outstanding patterns are in particular worth of being emphasized.

First, HCPs working in specialties of high complexity and/or high likelihood of emergency, besides having a greater propensity for IDM, also appear more likely to exhibit certain cognitive and behavioral tendencies during instances of IDM. As for the former (cognition), they tend rely to a greater extent on automatic, non-conscious pattern recognition, and they are more inclined to use affective signals (presentiments) as information for their decisions. As for the latter (behavior), they are more disposed to perform or request further medical investigation and/or additional treatment despite negative or inconclusive examination results, and they are less prone to deviate from the medical protocol. Furthermore, HCPs in specialties of high complexity seem more inclined (or compelled) to make intuitive diagnoses, whereas those working in specialties of high likelihood of emergency have a greater propensity for exercising exceptional vigilance over their patients and making rapid, intuitive treatment decisions.

Second, HCPs who have been formally educated in the subject of IDM appear more likely to exhibit certain cognitive and behavioral tendencies indicative of a better quality of IDM. In particular, they seem more disposed to perform or request further medical investigation and/or additional treatment, they are less prone to deviate from medical protocols or establish intuitive diagnoses, they have a greater tendency to consciously reflect on their mental processes, and they are more likely to base their decisions on the integration of different kinds of diagnostic and prognostic information acquired from multiple sources.

These results once again highlight the necessity of educating HCPs about the nature and the use of IDM in clinical practice. The primary consideration here is not so much to increase their willingness to engage in IDM, but to improve the quality of their intuitive decisions, i.e. to make sure they use their intuition in a safe and appropriate way, in accordance with the general principles and requirements of medical DM.

### Practical consequences and recommendations

It is now widely accepted among theoreticians that no single best way of DM exists in health care, and optimal results can only be achieved through a combination of CDM methods (Elstein, 2009; Marcum, 2012; Norman et al., 2007; Pelaccia et al., 2011). At an individual level this implies that clinicians should be equipped with a toolbox of analytical and non-analytical DM methods and they should be trained to choose the one best suited to each particular medical scenario (Bodemer et al., 2015; Stolper et al., 2011).

We propose that, going beyond the individual level, the whole system of health care management and education should aim to achieve a match between the job-specific factors calling for and the person-specific factors facilitating IDM. That is, acquiring the skills necessary for the appropriate use of intuition should be included in the training of HCPs, in particular for those intending to work in medical specialties of ‘high complexity’ and/or ‘high likelihood of emergency’.

Formal education could go a long way in this direction by introducing students to the nature and the appropriateness of IDM, teaching them about its risks and benefits, fostering students’ CR skills at a practical level, and helping them develop the meta-cognitive capacities necessary for becoming expert. As to the last point, various authors have emphasized the importance of expert HCPs’ ability to monitor their own cognitive processes and switch back and forth between analytical and non-analytical DM as necessary (Marcum, 2012; Pelaccia et al., 2011; Stolper et al., 2011). This means that being an expert clinician does not only require possessing a large experiential knowledge base allowing one to make medical decisions in a quick and automatic way, but also using one’s reflective ability to identify situations in which relying on intuition would be inadequate, and analytical processing is needed instead. This is a skill which could and should be taught to medical students.

Finally, developing HCPs’ interpersonal sensitivity should also be an important part of their training. Besides directly improving the human quality of patient-practitioner encounters, this would enable clinicians to be attuned to their patients in a way which is beneficial and often essential for gaining intuitive insights about their health conditions.

### Limitations and directions for further research

The most severe limitation of our research is that it relies solely on subjective, self-reported data about clinicians’ use of IDM. We cannot be sure to what extent the proportion of positive answers to the main question in our first survey reflected HCPs’ true propensity for IDM. So caution is required in interpreting our results, because any relationship we have found between respondents’ individual characteristics and their rate of positively answering the survey question could reflect a combination of effects on (1) clinicians’ true reliance on IDM; (2) their awareness of the fact that they do rely on IDM; (3) their propensity to be willing to admit this fact.

Another shortcoming is the oversimplified formulation of the main survey question, allowing only two response categories. Also, our definition of an ‘intuitive decision’ which we provided to survey participants was not quite sufficient. We could have measured more accurately the prevalence of intuitive instances in CDM by asking HCPs about how frequently and to what extent they relied on intuition in their decisions along with providing a sufficiently detailed explanation of IDM.

Concerning the generalizability of our findings, a further important issue is the quality and degree of representativeness of the sample which we collected for the first survey. We relied on chain-referral sampling, which, due to its uncontrolled nature, may have introduced imbalances to the data. Nonetheless, for physicians we were able to compare the sample with the population, and we found that on the main characteristics (gender, years of experience, medical specialty) its composition could be considered to a fair degree representative.

There are several venues for future research. Further investigation would be necessary regarding the appropriateness of IDM in distinctive medical specialties. It would be equally important to explore the intra-personal factors of HCPs’ reliance on IDM. These might involve the person’s general cognitive style as well as diverse aspects of his/her attitude to IDM. As a practical benefit, the key intra-personal factors of IDM thus identified could be used as targets of intervention in the process of selecting and/or training HCPs for medical specialties and positions in which intuitive practices are essential. This could potentially improve the quality of decisions in a range of medical fields.

## Data Availability

The data that support the findings of this study are openly available in Mandalay Data at https://doi.org/10.17632/ykmjj5bh4g

## Notes

1 In the ‘Results’ section we compare, on main characteristics, the composition of the sample with that of the target population.

2 See the next subsection.
